# Sleep problems and associated factors in children with juvenile idiopathic arthritis: a systematic review

**DOI:** 10.1186/1546-0096-12-19

**Published:** 2014-06-02

**Authors:** Jennifer N Stinson, Jill A Hayden, Sara Ahola Kohut, Charlene Soobiah, Jenny Cartwright, Shelly K Weiss, Manisha B Witmans

**Affiliations:** 1 Departments of Anesthesia and Pain Medicine, The Hospital for Sick Children, Toronto, Ontario, Canada; 2 Child Health Evaluative Sciences, The Hospital for Sick Children, Toronto, Ontario, Canada; 3 Lawrence S. Bloomberg Faculty of Nursing, University of Toronto, Toronto, Ontario, Canada; 4 Department of Community Health & Epidemiology, Dalhousie University, Halifax, Nova scotia, Canada; 5 Li Ka Shing Knowledge Institute, St. Michael’s Hospital, Toronto, Ontario, Canada; 6 Institute for Health Policy, Management & Evaluation, University of Toronto, Toronto, Ontario, Canada; 7 Department of Pediatrics, The Hospital for Sick Children, University of Toronto, Toronto, Ontario, Canada; 8 Department of Pediatrics, University of Alberta, Edmonton, Alberta, Canada

**Keywords:** Juvenile idiopathic arthritis, Sleep problems, Systematic review, Prognostic factor

## Abstract

**Background:**

Sleep problems are common among children with chronic illnesses such as Juvenile Idiopathic Arthritis (or JIA). However, little is known about the frequency and severity of sleep disturbance(s) and the factors that are associated with sleep problems in children with JIA. The mechanism(s) of the relationships characterizing the development or exacerbation of sleep problems in children with JIA are still unknown, however studies have reported an association. The purpose of this study was to synthesize existing research related to sleep problems in children with JIA.

**Methods:**

The Preferred Reporting Items for Systematic Reviews and Meta-analysis (PRISMA) statement guided the conduct and reporting of this review. An experienced librarian conducted searches in MEDLINE, EMBASE, PsychINFO, CINAHL, and the Cochrane Central Register of Controlled Trials from inception to January 2012, to identify potentially relevant citations. Two members independently selected, rated methodological quality using the QUIPS tool, and extracted data from included studies.

**Results:**

Ten studies were included and findings varied across studies; studies were mostly cross-sectional, or case-controlled designs, with only one cohort study available. Four studies found that children and adolescents diagnosed with JIA had significantly more sleep disturbances when compared to healthy controls. Pain was most often associated with sleep disturbances. The heterogeneous findings highlight the complex relationships between JIA and sleep, and low methodological quality of studies in the field.

**Conclusions:**

This review supports an association between poor sleep and increased symptoms related to JIA, specifically the experience of pain. However, results need to be interpreted cautiously given the inconsistent findings regarding factors associated with sleep problems in JIA, the limited evidence available, and its low quality. Furthermore it is not yet determined if the poor sleep patterns predate the symptoms reported with JIA. More research is vital to understanding the factors that predict or perpetuate poor sleep in children and adolescents diagnosed with JIA.

## Introduction

Sleep problems are common among children with chronic illnesses [1-3] such as Juvenile Idiopathic Arthritis (or JIA). JIA is one of the most common rheumatic diseases in childhood, affecting approximately 1 in 1000 children [4]. Findings suggest that fatigue is common [5] and that sleep is disrupted in children with JIA [6,7]. Adequate amount and quality of sleep is essential for normal child development [8,9]. Sleep disturbances collectively refer to impairments in the ability to initiate or maintain sleep, and can be measured by parent or child self-report and by objective measures such as actigraphy and polysomnography [10]. Some sleep disorders may affect a child’s daytime function, resulting in behavioural problems such as attention deficit, aggressiveness, hyperactivity, chronic fatigue, decrements in daytime alertness and performance, and an increase in school absenteeism [11-13]. Sleep disturbances have also been associated with children’s quality of life - negatively impacting children’s physical and emotional well-being [14,15].

Little is known about the frequency and severity of sleep disturbance(s) in children with JIA, although studies suggest that their sleep is disrupted [2,3]. Children with JIA and their parents report significantly more instances of night awaking, parasomnias, sleep-related anxiety, sleep-disordered breathing, early morning awaking and excessive daytime sleepiness than do healthy children [14]. Although sleep difficulties have been documented in children with arthritis, the underlying neuropsychophysiological and neurobiological mechanisms of sleep problems in JIA remain unclear [16]. Lewin and Dahl [17] hypothesize a bi-directional interplay between pain and sleep disturbance. However, there has been little or no analysis of other factors possibly contributing to these problems (physiological/disease related, psychological [cognitive/affective, behavioral], socio-cultural, social determinants of health), nor empirical investigations of sleep interventions in this population. Clinicians working with children with JIA, therefore, have little guidance to best treat or prevent sleep problems in this population, placing already vulnerable children at increased risk of experiencing the many negative outcomes associated with poor sleep, and potentially increasing their disease burden.

The purpose of this study was to synthesize existing research related to sleep in children with JIA to determine: (1) the most common sleep problems and their characteristics in children with JIA; (2) risk factors (e.g., physiological, disease-related, psychological and socio-cultural) associated with the onset of new sleep problems in children with JIA; and (3) prognostic factors (e.g., physiological, disease-related, psychological and socio-cultural) associated with the persistence of sleep problems in children with JIA. It is anticipated that information gleaned from this review will inform our understanding of potential associations and determinants of underlying sleep disturbances and also inform development of potential interventions to target these specific mechanisms.

## Review

We used the Preferred Reporting Items for Systematic Reviews and Meta-analysis (PRISMA) statement [18] to guide the conduct and reporting of this review. Studies reporting any sleep problems in children (ages 0-18 years) diagnosed with any subtype of JIA were included in the review.

### Electronic database search

An experienced librarian (Nickle) conducted a search in MEDLINE, EMBASE, PsychINFO, CINAHL, and the Cochrane Central Register of Controlled Trials from inception to January 2012, to identify potentially relevant citations. A full search strategy in MEDLINE is available on request from authors. Search strategies were conducted with no restrictions by language or year of publication. The reference lists of all included studies were scanned to identify further studies.

### Study selection and characteristics

Studies reporting sleep problems in children (ages 0-18 years) diagnosed with any subtype of juvenile idiopathic arthritis (JIA) or juvenile rheumatoid arthritis (JRA) [19] were included in the review. To avoid potential confounding, studies including children with both JIA or JRA and also co-morbid psychiatric disorders were excluded. Studies were included if they investigated factors associated with the severity or frequency of sleep problems. Sleep problem was defined as any of the following: 1) sleep disorder (as defined by the International Classification of Sleep disorders ICSD-2), 2) manifestations of sleep problems such as difficulty initiating or maintaining sleep, or 3) inadequate sleep duration as defined by study authors. All study designs were included in the review, such as experimental, quasi-experimental and observational studies. Studies not reporting original data, case studies, letters to the editor without original data and commentaries were excluded. Due to resource constraints, non-English studies were excluded from the review. Two reviewers (CS, JB) independently screened all titles and abstracts. Potentially relevant full text articles were assessed for inclusion using an agreed upon eligibility form developed a priori. Discrepancies were resolved by group discussion with team members (JS, JH, CS, JB).

### Data abstraction

A draft data abstraction form was developed and pilot tested in Microsoft Excel 2007 version 12 [20]. Two reviewers (CS, JB) extracted data from included articles independently and any discrepancies in data extracted were resolved by group discussion.

### Methodological quality assessment

Methodological quality was assessed using the QUIPS tool [21,22], which assesses risk of bias (ROB) in prognostic factor studies. (Additional file 1). Two reviewers (CS, JC) independently assessed each study for ROB based on information available in the respective manuscripts. No attempts were made to contact authors to retrieve additional information on ROB.

### Synthesis

Due to heterogeneity across studies, a meta-analysis could not be conducted. The literature review results and synthesis of studies is described narratively and the strength of evidence for each type of prognostic factor is summarized using the following guidelines:

1) “**Strong evidence of effect:** Consistent findings (defined as > 75% of studies showing the same direction of effect) in multiple low ROB studies;

2) **Moderate evidence of effect:** Consistent findings in multiple high ROB and/or one study with low ROB;

3) **Limited evidence of effect:** One study;

4) **Conflicting evidence:** Inconsistent findings across studies; and

5) **No evidence:** No association between prognostic factor and the outcome of interest [23]”.

Inter-rater agreement was assessed using a weighted Cohen’s Kappa. Interpretation of kappa statistic was based on established categorization: poor (K <0.00), slight (0-.00-0.20), fair (0.21-0.40), moderate (0.41-0.60), substantial (0.61-0.80) and almost perfect (0.81-1.00) [23,24].

## Results

**Figure 1:**
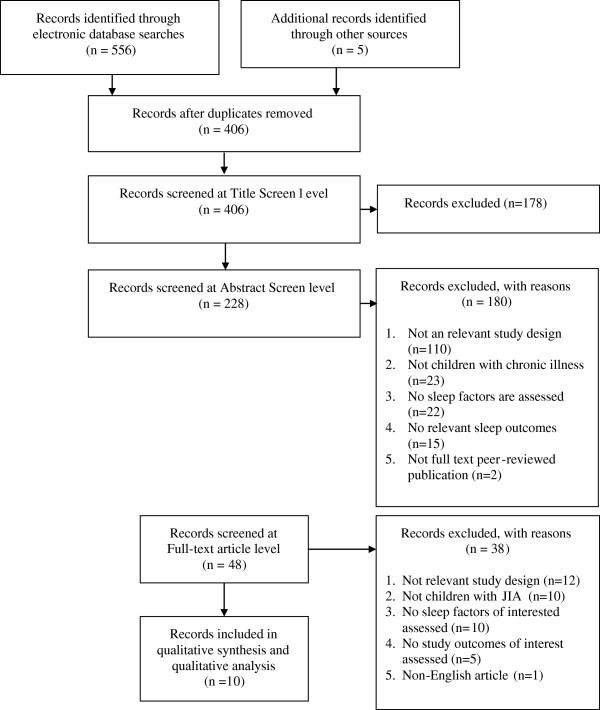
Study flow.

The literature search yielded 406 independent titles and abstracts, of which 10 studies fulfilled eligibility criteria and were included in the review [1,3,7,25-31] (see Figure 1). Reliability between reviewer assessments were considered moderate (K = 0.501).

### Patient characteristics of included studies

All of the studies were conducted in clinical settings. Diagnosis of JIA/JRA was variable across studies and many used various criteria, including those of the American College of Rheumatology [7,25,27], the International League of Associations for Rheumatology (ILAR) [3,29,31] or the Durban criteria [31]. Two studies distinguished between active and inactive JIA [28,30] and one study did not specify how children were diagnosed with JIA [1]. Half of the included studies excluded participants if they had a preexisting or family history of sleep problems [2,3,27,28,30]. One study included participants with a family history of sleep problems [31], and one study explicitly stated inclusion of participants without regard to sleep problems [7]. The remaining studies did not report on inclusion of sleep problems in JIA population.

Sample size of the included studies ranged from 20 to 122 patients, and females were more represented than males. The average age of patients was 11.45 years and ages ranged from 6-17 years (Table 1). The average duration of JIA/JRA was 3.8 year and ranged from 3.4-4.5 years, however disease duration was only reported in a few studies [3,7,25,26]. The most prevalent JIA subtypes were oligoarticular and polyarticular; these subtypes were represented in all of the included studies. Enthesitis-related arthritis (ERA) was the least common JIA subtype and was represented in only one study [31] (Table 1).

**Table 1:** Study and patient characteristics

**Author (Year)**	**Country**	**Study design**	**Recruitment setting**	**Sample size (numbers)**	**Mean age (SD)**	**% Female**	**JIA/JRA diagnosis**	**Disease duration**
								**Mean Year (SD)**
Zamir (1998) [7]	Israel	Case control	Pediatric rheumatology clinic	JRA: 16	12 (4)	69	Olig: 4	4.5 (3.4)
				Controls: 9			Poly: 12	
Bloom (2002) [25]	USA	Case control	Hasbro Children's Hospital, Pediatric Rheumatology Clinic	JRA: 25	8.7	80	Syst: 5	3.1 (range: 0.2-5.5 )
				Controls: 45	Range: 6-12		Olig: 9	
							Poly: 11	
Palermo (2005) [26]	USA	Cross- sectional	Outpatient pediatric rheumatology clinics (NS)	JIA: 20	NR	67	Syst: 2	4.49 (NR)
							Olig: 5	
							Poly: 7	
							ERA: 2	
							NS: 4	
Passarelli (2006) [27]	Brazil	Case-control	NR	JRA: 21	13 (2)	57	Syst: 9	NR
				Controls: 20			Poly: 12	
Long (2008) [1]	USA	Cross sectional	Four clinical sites during specialty care visits (NS)	JIA: 30	Range: 8-12	56	Syst: 3	NR
							Olig:21	
							Poly: 6	
Ward (2008) [3]	USA	Cross sectional	Children's Hospital and Regional Medical Centre- Paediatric Rheumatology Clinic	JRA: 70	8.5 (1.9)	84	Syst: 4	Inactive JRA: 3.8 (1.7)
							Olig: 26	
							Poly: 40	Active JRA: 3.4 (3.0)
Ward (2010) [28]	USA	Cross sectional	Seattle Children's Hospital and University of Washington School of Nursing sleep laboratory	JIA: 69	8.5 (1.9)	84	Sys: 3	NR
							Olig: 26	
							Poly: 40	
Butbul Aviel (2011) [29]	Canada	Cross sectional	The Hospital for Sick Children	JIA: 92	12.7 (0.4)	66	Syst: 28	NR
							Olig: 31	
							Poly: 33	
Ward (2011) [30]	USA	Case control	Seattle Children's Hospital Paediatric Rheumatology Clinic	JIA: 70	8.5 (1.9)	76	Sys: 4	NR
				Controls: 46			Olig: 26	
							Poly: 40	
Bromberg (2012) [31]	USA	Cohort	Pediatric Rheumatology Clinic	JIA: 51	12.4 (2.8)	65	Poly: 51	NR

*Abbreviations: *ERA* enthesitis-related, *JIA* juvenile idiopathic arthritis, *JRA* juvenile rheumatoid arthritis, *NS* not specified, *NR* not reported, *Olig* oligoarticular, *Pauc* pauciarticular, *Poly* polyarticular, *RCT* randomized controlled trial, *SD* standard deviation, Sys systemic arthritis.

### Methodological quality of studies

Study participation was deemed adequate except in three studies [3,28,30] where ROB was moderate (Table 2). These studies used convenience samples and may not be representative of JIA/JRA patients as a whole. Low ROB was observed due to prognostic factor measurement and due to statistical analysis and reporting across all studies. We rated two studies as having a moderate ROB [7,27] and two studies as high ROB due to potential confounding [25,29] (Table 2). Confounding variables such as age and gender were not accounted for in two studies and contributed to a greater ROB [7,27].

**Table 2:** Risk of bias

**Author (YR)**	**Level of risk of bias due to study participation**	**Level of risk of bias due to study attrition**	**Level of risk of bias due to prognostic factor measurement**	**Level of risk of bias due to confounding measurement and account**	**Level of risk of bias due to analysis**	**Outcome assessed**	**Level of risk of bias due to outcome measurement**
Zamir (1998) [7]	Low	N/A	Low	High	Low	Sleep fragmentation	Low
Bloom (2002) [25]	Low	N/A	Low	Moderate	Low	CHSQ total score	Low
						SSR score	Moderate
Palermo (2005) [26]	Low	N/A	Low	Low	Low	Sleep/Wake Behaviour Problems	Low
Passarelli (2006) [27]	Low	N/A	Low	High	Low	Sleep disorders	Low
Long (2008) [1]	Low	N/A	Low	Low	Low	Sleep disturbance	Low
Ward (2008) [3]	Moderate	N/A	Low	Low	Low	Sleep disturbance	Low
						Sleep quality	Low
Ward (2010) [28]	Moderate	N/A	Low	Low	Low	Number of wake bouts	Low
						Arousals	Low
						Apnea/Hypopnea Index (AHI)	Low
Butbul Aviel (2011) [29]	Low	N/A	Low	Moderate	Low	CSHQ sleep disturbance	Low
						SSR sleep disturbance	Moderate
Ward (2011) [30]	Moderate	N/A	Low	Low	Low	Total Sleep Disturbance score	Low
Bromberg (2012) [31]	Low	Low	Low	Low	Low	Sleep quality	Low

*Abbreviations: *CHSQ* Children's Sleep Habits Questionnaire, *SSR* sleep self report. *Note. N/A* not applicable.

### Sleep disturbances in children with JIA

Four studies examined and compared sleep disturbances in children with JIA/JRA and healthy controls [7,25,27,30] (Table 3). Two studies found that when JRA patients were compared to healthy controls, they had shorter stage 2, stage 3 and REM sleep stages (p < 0.05)^7^ and higher indexes of periodic leg movements, arousals and alpha activity in non-REM sleep (p < 0.01) [27]. Two studies compared JRA and JIA patients to healthy controls using the Children’s Sleep Habits Questionnaire (CSHQ) [25,30]. Both studies found that JIA and JRA patients had significantly greater overall sleep disturbances, particularly in the areas of night wakings, parasomnias, sleep anxiety, sleep disordered breathing, and daytime sleepiness (all p <0.05) [25,30]. One study found no significant differences in reaction time between JIA patients and healthy controls as measured by performance on the Cambridge Neuropsychological Test Automated Battery (CANTAB) [31]. Overall, there is evidence to suggest that children and adolescents with JIA/JRA have increased disturbances in sleep wake patterns and behaviours when compared to healthy controls.

**Table 3:** Studies investigating sleep problems in JIA compared to healthy controls

**Author (YR)**	**Sleep domains examined**	**Measurement of sleep outcome**	**Factors examined**	**Measurement of factor**	**Results**
**Sleep wake patterns and behaviours**					
Zamir (1998) [7]	Total number of index arousals, or, stage shifts or leg movements (Sleep fragmentation)	Polysomnography	Number of active joints, Duration of stiffness, ESR	Rheumatologic examination	JRA patients had more arousals and awakenings per hour compared to controls (22.7 ± 9.6 vs 11.7 ± 5.3, p < 0.001)
					Median length of stage 2 sleep was 60% shorter in JRA (8.0 mins) compared to controls (13.5 mins) (p < 0.001)
					Median length of stage 3 sleep was also shorter in JRA patients (5.8 mins) compared to controls (9.8 mins) (p < 0.001)
					Median length of REM sleep was also shorter in JIA patients (9.4 mins) compared to controls (15.1 mins) (p < 0.02)
Bloom (2002) [25]	Sleep habits	CSHQ			JRA patients compared to controls had a high total score on the CSHQ (45.36 ± 8.01 vs 37.51 ± 6.12, p < 0.0001), as well as higher scores on the following subscales: night wakenings (4.56 ± 1.29 vs 3.42 ± 0.82, p < 0.001), parasomnias (10.0 ± 1.85 vs 8.19 ± 1.11, p < 0.001), sleep anxiety (5.63 ± 1.61 vs 4.89 ± 1.26, p = 0.045), sleep-disordered breathing (3.70 ± 1.02 vs 3.25 ± 0.53 p = 0.036), and morning wakening/daytime sleepiness (11.88 ± 2.80 vs 9.91 ± 2.68, p = 0.007)
Passarelli (2006) [27]	Alpha/delta waves, periodic leg movement, isolated leg movements	Polysomnography	Pain score	Self-assessment of pain on a categorical 5-point face scale ranging from “no hurt” to “hurts worst”	JRA patients exhibited higher indexes of periodic leg movements (p = 0.02), isolated leg movements, and arousals, as well as increases in alpha activity in non-REM sleep (all p < 0.01), in spite of similar frequency of sleep complaints in comparison to controls
Ward (2011) [30]	Sleep disturbance	CSHQ			JIA patients compared to controls had a statistically significant (P < 0.001) greater mean overall sleep disturbance score (45.0 ± 7.3 vs 39.1 ± 4.9) and higher scores on 6 of 8 subscales including; sleep onset delay (1.6 ± 0.7 vs 1.2 ± 0.4, p = 0.001), sleep anxiety (5.8 ± 2.0 vs 5.0 ± 1.4, p = 0.02), night wakenings (4.0 ± 1.3 vs 3.3 ± 0.7, p = 0.001), parasomnias (9.3 ± 1.8 vs 7.9 ± 1.1, p = 0.001), sleep disordered breathing (2.3 ± 0.6 vs 2.1 ± 0.4, p = 0.03), and daytime sleepiness (13.0 ± 3.5 vs 11.0 ± 3.3, p = 0.004)
			Reaction time	CANTAB	There were no group differences on neurobehavioral performance test scores.

*Abbreviations*: *ESR* erythrocyte sedimentation rate, *JRA* Juvenile rheumatoid arthritis, *CSHQ* child sleep habits questionnaire.

### Prognostic factors associated with sleep outcomes examined

In total, six studies examined disease-related factors associated with persistence of sleep problems [3,7,25,27,29,31], and all disease-related factors were gathered data from rheumatologic examinations.

Seven studies examined psychological-related factors associated with sleep disturbance [1,3,25,26,28-30], all of which were measured using validated scales (Table 4).

**Table 4:** Studies investigating relationship between prognostic factors and sleep outcomes

**Author (YR)**	**Sleep domains examined**	**Measurement of sleep outcome**	**Factors examined**	**Measurement of factor**	**Results**
**Sleep wake patterns and behaviours**					
Zamir (1998) [7]	Total number of index arousals, or, stage shifts or leg movements (Sleep fragmentation)	Polysomnography	Number of active joints, Duration of stiffness, ESR	Rheumatologic examination	Multiple linear regression revealed no association between number of active joints, duration of stiffness, or ESR, with the total number or index of arousals or awakening, stage shifts, or leg movements (NS)
Palermo (2005) [26]	Sleep wake problems	Sleep-Wake Behavior Problems Scale	Functioning	FDI	In multivariate regression functioning was predictive of sleep wake problems (β = 0.665, p = 0.054)
			Pain severity	Faces Pain Scale	In multivariate regression pain severity was not significantly predictive of sleep wake problems (β = 0.593, p = 0.126)
			Pain frequency	6-point scale ranging from less than once a month to daily	In multivariate regression, pain frequency was not significantly predictive of sleep wake problems (β = -0.162, p = 0.665)
Passarelli (2006) [27]	Alpha/delta waves, periodic leg movement, isolated leg movements	Polysomnography	Morning stiffness	Rheumatologic examination	Morning stiffness was significantly correlated to periodic leg movement (r_s_ = 0.75, p = 0.00009) and isolated leg movements (r_s_ = 0.78, p = 0.00003)
			Pain score	Self-assessment of pain on a categorical 5-point face scale ranging from “no hurt” to “hurts worst”	Pain score was significantly correlated with alpha/delta waves (r_s_ = 0.74, p = 0.0001)
Ward (2008) [3]	Wake and sleep stages, apnea/ hypopnea index (AHI), periodic leg movements	Polysomnography	Sleep quality	SSR	In the multivariate regression model testing predictors of the disturbed sleep (arousals), age and medications, anxiety, and evening pain explained 18% of variance, but neither anxiety or pain had a significant effect (both p > .05)
			Anxiety	RCMAS	Anxiety did not predict sleep disturbances (β = -0.30, p = 0.19)
			Medications	Parents completed a daily diary of medications their child received	Medications did predict sleep disturbance (β = 0.11, p < .04)
			Evening pain	Oucher Faces Rating Pain Scale	Evening pain did not predict sleep disturbances (β = 0.23, p = 0.19)
Ward (2010) [28]	Apnea/ hypopnea index (AHI), awakenings, arousal	Polysomnography	Reaction time	CANTAB	Reaction time was inversely correlated with awakenings and arousals (r = -0.32, p < 0.03)
**Inadequate sleep quality**					
Bloom (2002) [25]	Sleep habits	CSHQ	Function	JAFAR	Functional disability was not significantly correlated with sleep habits (r_s_ = 0.253, p = 0.222)
			Limited joint count	NR	Limited joint count was not significantly correlated with sleep habits (r_s_ = -0.184, p = 0.380)
			Active joint count	NR	Active joint count was not significantly correlated with sleep habits (r_s_ = -0.100, p = 0.633)
			Parent global rating	Varni Pediatric Pain Questionnaire	Parental global rating was not significantly correlated with sleep habits (r_s_ = 0.262 p = 0.207)
			Physician global rating	Overall disease activity on a scale of 0-4 (0 = no disease activity, 4 = very severe disease)	Physician global rating was not significantly correlated with sleep habits (r_s_ = 0.258, p = 0.212)
			ESR	Clinical pathology laboratory by standard methods	ESR was not significantly correlated with sleep habits (r_s_ = 0.102, p = 0.628)
		SSR	Average pain	VAS	Average pain score was significantly correlated with sleep habits (r_s_ = 0.56, p = 0.005)
Long (2008) [1]	Sleep disturbance	CSHQ	Functioning	FDI - child and parent report	Child report of functional disability was not significantly correlated with sleep disturbance (r = 0.190, NS)
					Parental report of functional disability was significantly correlated with sleep disturbance (r = 0.646, p < 0.01)
			Physical and psychosocial HRQOL	Child’s Health Questionnaire	Physical and psychosocial HRQOL was inversely correlated with sleep disturbance (r = -0.813, p < 0.01)
			Disease severity (global rating), daily pain	VAS (100-mm)	Disease severity was significantly correlated with sleep quality (β = 0.05, p > .05)
Butbul Aviel (2011) [29]	Sleep disturbance	CSHQ	Number of tender and swollen joints	Number of swollen and painful joints by parents’ and patients’ self-report joint count—using a pictorial (mannequin) format.	Self reported sleep habits was slightly correlated with number of tender joints (r = 0.241) and swollen joints (r = 0.163)
			Global pain, worst pain	VAS	Self reported sleep habits was significantly correlated with global pain (r = 0.32, p = 0.0003)
			Number of painful areas, present pain	SSR	Self reported sleep habits was significantly correlated with (r = 0.32, p = 0.0003)
			Fatigue	PedsQL fatigue	Self reported sleep habits were inversely correlated with self reported fatigue (r = -0.45, p < 0.0001)
Ward (2011) [30]	Sleep disturbance	CSHQ	Reaction time	CANTAB	Reaction time on CANTAB was significantly correlated with sleep disturbance (β = 0.18, p = 0.22)
Bromberg (2012) [31]	Sleep quality	VAS (100-mm, ranging from did not sleep well to slept very well)	Age		Age was inversely correlated with sleep quality (β = -0.39, p > .05)

*Abbreviations*: *CANTAB* Cambridge neuropsychological test automated battery, *CSHQ* child sleep habits questionnaire, *ESR* erythrocyte sedimentation rate, *FDI* functional disability inventory, *HRQOL* health related quality of life, *JAFAR* Juvenile arthritis functional assessment report, *JRA* Juvenile rheumatoid arthritis, *NR* not reported, *PedsQL* fatigue pediatric quality of life inventory multidimensional fatigue scale™, *RCMAS* revised children’s manifest anxiety scale, *SSR* sleep self report, *VAS* visual analogue scale.

Several studies examined the relationship between sleep disturbance and negative physical and psychological factors [1,30,31]. Pain was assessed using a variety of pain scales [32-35]. Only one study examined demographic factors and included age as a potential prognostic factor [26] (Table 4).

### Prognostic factors associated with sleep-wake patterns and behaviours

In total, five studies provided evidence on prognostic factors associated with sleep-wake patterns and behaviours in children with JRA or JIA [3,7,26-28]. One study found no significant associations for number of active joints, duration of stiffness or ESR when compared with total number or index of arousals or awakening, stage shifts, or leg movements^7^. However, in another single study, morning stiffness was found to be correlated with periodic leg movements and isolated leg movements (p < 0.001) [27]. The same study found that self-reported pain scores were correlated with alpha and delta sleep waves (p < 0.01, Table 4) [27]. It was not clear from any of these studies, whether participants were in a flare or not which might impact sleep-wake patterns.

Self-reported functional disability was associated with sleep-wake problems (p < 0.05), however pain severity and frequency were not found to be related in the same study [26]. Other potential prognostic factors including pain severity, pain frequency, evening pain, medications, and anxiety did not appear to be related to sleep wake patterns [3,26,27] (Table 4). One study reported that reaction time on the CANTAB was inversely related to awakenings and arousals (p < 0.03) [28].

### Prognostic factors associated with inadequate sleep quality

Five studies examined prognostic factors associated with sleep quality, of which four used the CSHQ [1,25,29,30] and one used a modified VAS (Visual Analogue Scale) for sleep quality [25]. Two studies looked at the relationship between functional disability and sleep habits, and found that self-reported functioning was not correlated with sleep disturbance [1,20], however, parental reporting of functional disability was significantly correlated (p < 0.01, Table 4)^1^.

Bloom and colleagues found that disease-related factors, limited joint count, active joint count ESR, were not significantly correlated with sleep quality [25]. Another study found swollen joints and tender joints to be slightly correlated to sleep disturbance (r = 0.24, and 0.16 respectively, Table 4) [29].

One study found parental and physician global ratings for pain were not significantly associated with sleep habits (p = 0.2) [25]. Three studies found that self-reported severity and frequency of pain were significantly related to sleep disturbance and quality [25,29,31].

Only one study examined age in relation to sleep problems and found that age was inversely correlated to sleep disturbance (p < 0.01)^1^. Physical and psychosocial health-related quality of life was also inversely correlated with sleep disturbance (p < 0.01)^1^. Conversely, reaction time on the CANTAB was not found to be related to sleep disturbance (p = 0.22) [30].

## Conclusions

Sleep disturbances are a common challenge in children and adolescents with chronic illness and can negatively impact physical and psychosocial health-related quality of life [36]. This study aimed to systematically review evidence about sleep disturbances in children diagnosed with JIA, and associated prognostic factors. Only ten studies were identified and findings varied across studies. Studies were mostly cross-sectional, or case-control study designs, with only one prospective longitudinal study available. Inconsistent findings highlight the complex relationships between JIA and sleep, and may be due to low methodological quality of studies (e.g., not rigorous longitudinal study design, moderate ROB due to study participation and confounding variables) currently available in the field. In addition, previous research may not be measuring the appropriate outcomes when using a PSG and actigraphy (e.g., research may benefit from measuring neurometabolites important in the regulation of the circadian rhythm of sleep, such as melatonin and cortisol levels as well as other metabolites common to pain and sleep such as inflammatory cytokines to examine whether there is a difference) [3,7,25,27-30]. Despite limited number of high quality studies, the results of this review suggest that children and adolescents diagnosed with JIA appear to have significantly more sleep disturbances when compared to healthy controls [7,25,27,30]. By self-report, pain was commonly associated with various aspects of sleep disturbances, however pain was not found to be associated directly with sleep disturbances [25-27,29]. The lack of association may be the result of the different types of sleep problems that occur in children, which may or may not be attributable to JIA/JRA. The available evidence provided no consistent findings about other disease-related factors being associated with sleep disturbances. Several explanations for this may include the age of the children, the type of measurements tools that were used, and the relatively small sample sizes. These findings highlight the limitations of the available literature in determining the direction of effect between potential prognostic factors and outcomes, and remaining potential for confounding due to known and unknown factors.

Several potential mechanisms have been hypothesized as causing sleep problems in children resulting from any combination of physiological, psychological, and behavioural alterations. Physiological mechanisms have been proposed, suggesting sleep problems are the result of disturbances in the neural circuitry and neurotransmitters that underlie sleep and circadian rhythms, such as alteration of GABA or dopamine [37]. Alternatively, there are questions about whether poor sleep regulation early in infancy or childhood prime the circuitry with increased auto-immune dysregulation such that these children are predisposed to JIA. There are some emerging data in mental health to suggest that sleep may affect the neural circuitry to predispose or perpetuate mental disorders and whether this could be true for pain-related conditions remains to be determined [38]. Our study did not include children with associated comorbid psychiatric or mental health issues. Other potential mechanisms may relate to emotional (e.g., anxiety) or behavioral dysregulation (e.g., bedtime refusal), interfering with sleep or further exacerbating underlying sleep disruption [11,39].

Previous studies of sleep in children with arthritis suggested pain as the cause of sleep problems [25,27], and no other disease-specific variables have been examined as predictors of sleep problems. However, conceptual models of sleep and pain suggest a complex, bidirectional relationship [17,40]. A recent systematic review focused on sleep disturbances in pediatric pain populations (including children with JIA) also proposed that the bidirectional relationship between sleep and pain interacts with physiological and psychological factors to influence functional outcomes [40]. The findings of this review suggest that sleep may play a more significant role in predicting pain as opposed to a bidirectional relationship, but this finding remains unclear [40]. To build upon these findings, the current review included all disease-related variables (in addition to pain) as potential factors influencing sleep disturbances in pediatric JIA samples.

**Figure 2:**
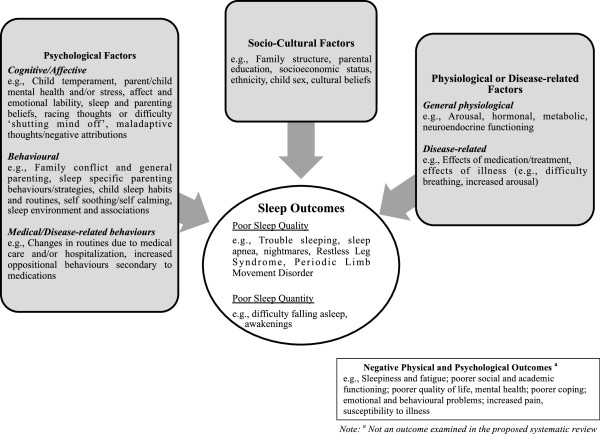
Model of sleep problems in healthy children with potential adaptations for children with chronic illnesses.

Overall, the heterogeneous results of the current review not only reflect the complex inter-relationships between pain and sleep but more broadly between JIA and sleep, as well as the need for high quality longitudinal studies in the field. Figure 2 depicts a proposed model of sleep disturbances in children and adolescents with chronic illness, such as JIA. This model was originally developed to illustrate sleep problems in healthy children [41-44] and was subsequently adapted for children with chronic illnesses. The model depicts theoretical relationships among physiological, disease-related, psychological (cognitive/affective/behavioural) and socio-cultural factors (including social determinants of health) that may contribute to the development and maintenance of sleep problems among children with chronic illnesses. These are relationships that have not been systematically established in children with chronic illnesses, but that we may expect to find, given existing knowledge of sleep in healthy children. Potential factors associated sleep problems that may be unique to children with chronic illnesses (e.g., effects of medication) are depicted in grey. Briefly, socio-cultural, psychological, physiological and disease-related factors may influence children’s ability to sleep at night, partially through their effects on children’s physiology (e.g., arousal). Sleep-wake patterns and behaviours may become disturbed, indicating poor sleep quality, and leading to inadequate sleep quantity. For some children, sleep disorders may develop as a result of this process. For others, sleep disorders may be pre-existing or co-morbid, furthering impairing sleep. In the final stage of this model, inadequate sleep is depicted as resulting in negative physical and mental health outcomes.

### Future directions for research

Further research is necessary to validate and adapt our proposed model of sleep disturbances in children and adolescents with JIA. Future longitudinal studies are necessary to identify specific factors predicting sleep disturbances in youth with JIA as well as to clarify the direction of the relationship between sleep disturbances and pain. Determining specific factors related to quality and quantity of sleep would allow for the development of appropriate physiological, psychological, and socio-cultural interventions to address sleep disturbances in this vulnerable population. Sleep interventions may in turn buffer the effects of poor sleep on chronic illness and help alleviate suffering in this vulnerable population.

In summary, findings of this review indicate that children and adolescents diagnosed with JIA have significantly more sleep disturbances compared to healthy peers. Support is given to the negative influence of poor sleep on health-related quality of life as well as specifically on the experience of pain. However, results of this review need to be interpreted cautiously given the inconsistent findings regarding factors associated with sleep problems in JIA, the limited evidence available, and its low quality. More research is vital to developing an understanding of the factors that predict poor sleep in children and adolescents diagnosed with JIA.

## Competing interests

The authors declare that they have no competing interests.

## Authors’ contributions

JNS and JAH were involved in conceptualization, design, analysis, interpretation and manuscript preparation. SAK was involved in analysis, interpretation and manuscript preparation. CS was involved in screening citations and full text, data abstraction, risk of bias, and manuscript preparation. JC was involved in data abstraction and risk of bias. SKW and MBW were involved in interpretation, manuscript editing, and provided clinical expertise. All authors have edited and approved the final manuscript.

## Supplementary Material

Additional file 1 Quality in Prognostic Studies (QUIPS) tool. Click here for file
